# First Report on Chitin in a Non-Verongiid Marine Demosponge: The *Mycale euplectellioides* Case

**DOI:** 10.3390/md16020068

**Published:** 2018-02-20

**Authors:** Sonia Żółtowska-Aksamitowska, Lamiaa A. Shaala, Diaa T. A. Youssef, Sameh S. Elhady, Mikhail V. Tsurkan, Iaroslav Petrenko, Marcin Wysokowski, Konstantin Tabachnick, Heike Meissner, Viatcheslav N. Ivanenko, Nicole Bechmann, Yvonne Joseph, Teofil Jesionowski, Hermann Ehrlich

**Affiliations:** 1 Institute of Chemical Technology and Engineering, Faculty of Chemical Technology, Poznan University of Technology, Berdychowo 4, 61131 Poznan, Poland; soniazolaks@gmail.com (S.Ż.-A.); marcin.wysokowski@put.poznan.pl (M.W.); teofil.jesionowski@put.poznan.pl (T.J.); 2 Institute of Experimental Physics, TU Bergakademie-Freiberg, Leipziger str. 23, 09559 Freiberg, Germany; iaroslavpetrenko@gmail.com; 3 Natural Products Unit, King Fahd Medical Research Center, King Abdulaziz University, Jeddah 21589, Saudi Arabia; lshalla@kau.edu.sa; 4 Suez Canal University Hospital, Suez Canal University, Ismailia 41522, Egypt; 5 Department of Natural Products, Faculty of Pharmacy, King Abdulaziz University, Jeddah 21589, Saudi Arabia; dyoussef@kau.edu.sa (D.T.A.Y.); ssahmed@kau.edu.sa (S.S.E.); 6 Department of Pharmacognosy, Faculty of Pharmacy, Suez Canal University, Ismailia 41522, Egypt; 7 Leibniz Institute of Polymer Research Dresden, 01069 Dresden, Germany; tsurkan@ipfdd.de; 8 P.P. Shirshov Institute of Oceanology of Academy of Sciences of Russia, 117997 Moscow, Russia; tabachnick@mail.ru; 9 Faculty of Medicine Carl Gustav Carus, Dresden University of Technology, 01307 Dresden, Germany; Heike.Meissner@uniklinikum-dresden.de; 10 Department of Invertebrate Zoology, Biological Faculty, Lomonosov Moscow State University, 119992 Moscow, Russia; ivanenko.slava@gmail.com; 11 Institute of Clinical Chemistry and Laboratory Medicine, University Hospital Carl Gustav Carus, Medical Faculty Carl Gustav Carus, Technische Universität Dresden, 01307 Dresden, Germany; nicole.bechmann@uniklinikum-dresden.de; 12 Institute of Electronics and Sensor Materials, TU Bergakademie Freiberg, 09599 Freiberg, Germany; yvonne.joseph@esm.tu-freiberg.de

**Keywords:** Porifera, Demosponges, *Mycale*, chitin, sponge skeleton

## Abstract

Sponges (Porifera) are recognized as aquatic multicellular organisms which developed an effective biochemical pathway over millions of years of evolution to produce both biologically active secondary metabolites and biopolymer-based skeletal structures. Among marine demosponges, only representatives of the Verongiida order are known to synthetize biologically active substances as well as skeletons made of structural polysaccharide chitin. The unique three-dimensional (3D) architecture of such chitinous skeletons opens the widow for their recent applications as adsorbents, as well as scaffolds for tissue engineering and biomimetics. This study has the ambitious goal of monitoring other orders beyond Verongiida demosponges and finding alternative sources of naturally prestructured chitinous scaffolds; especially in those demosponge species which can be cultivated at large scales using marine farming conditions. Special attention has been paid to the demosponge *Mycale euplectellioides* (Heteroscleromorpha: Poecilosclerida: Mycalidae) collected in the Red Sea. For the first time, we present here a detailed study of the isolation of chitin from the skeleton of this sponge, as well as its identification using diverse bioanalytical tools. Calcofluor white staining, Fourier-transform Infrared Spcetcroscopy (FTIR), electrospray ionization mass spectrometry (ESI-MS), scanning electron microscopy (SEM), and fluorescence microscopy, as well as a chitinase digestion assay were applied in order to confirm with strong evidence the finding of a-chitin in the skeleton of *M. euplectellioides*. We suggest that the discovery of chitin within representatives of the *Mycale* genus is a promising step in their evaluation of these globally distributed sponges as new renewable sources for both biologically active metabolites and chitin, which are of prospective use for pharmacology and biomaterials oriented biomedicine, respectively.

## 1. Introduction

The structural polysaccharide chitin exists as a dominant component in the skeletal structures of diverse fungi [1,2,3], diatoms [4], sponges [5,6,7,8,9], corals [10], mollusks [11,12], annelids [13] and arthropods (see for review [14]). This very ancient biopolymer generally occurs in association with different kinds of organic biomacromolecules (pigments, lipids, other polysaccharides and proteins), as well as with calcium- and silica-based biominerals [15]. Interaction between chitin and the other organic and inorganic components listed above provides rigidification to a broad variety of invertebrate skeletal constructs. Mechanical stiffness of skeletons are also of crucial importance in sponges (Porifera), where chitin was recently reported in both marine (see for review [16,17]) and fresh water [18,19] species representing the class Demospongiae. In some sponges, chitin has been suggested as a template for formation of biomineralized structures in form of aragonite-silica-chitin [20], or silica-chitin [6,21], composites.

Analysis of the structural and physicochemical properties of this amino polysaccharide can be performed using a range of modern instrumentation [22]; and several review articles covering this biopolymer have been published recently [23,24,25]. There are no doubts that chitin (even without its derivative chitosan) is still of interest for applications as an adsorbent [23,26] and biomaterial for biomedical aims; for example, reconstruction of peripheral nerves or wound management [24,25,27]. It is worth noting here that thus far, only chitin of fungal and arthropod origins can be isolated on industrial scales. However, only sponges produce tube-like, fibrous three-dimensional (3D) chitinous scaffolds which are originally macroporous due to their basic role in the skeletons of these filter-feeder organisms. Chitin of demosponge origin in particular resembles the shape of the living sponges [9]. This phenomenon was also observed in the Cambrian fossil demosponges, *Vauxia gracilenta* [28]. The mechanical and thermal stability of chitinous scaffolds are key to their successful applications in modern biomimetics [29,30,31,32,33,34] and tissue engineering [8,35,36]. However, these achievements have been based exclusively on chitin isolated from diverse representatives of only one order of marine demosponges, the order Verongiida. We even suggested that the presence of chitin within skeletons of demosponges is unique for the order Verongiida only, and, consequently, the proposal to use this feature as diagnostic tool for systematics of all sponges related to the order Verongiida arose. Our single doubt was based on the finding of chitin in fresh water non-verongiid demosponges [18,19] (Spongillida) and, definitively, derived from marine sponges in ancient times.

Consequently, two years ago we started an intensive study of diverse non-verongiid marine demosponges with the aim to purify and identify chitin from other taxa of marine sponges. Especially, we have taken advantage of the worldwide distribution of the members of the genus *Mycale* [37] (Demospongiae: Heteroscleromorpha: Poecilosclerida: Mycalidae) living in a considerable depth range from intertidal to abyssal depth [38,39,40,41,42,43,44,45,46,47,48,49,50]. About 251 valid species belong to the genus *Mycale* are currently accepted from a pool of more than 500 nominal names [49,51,52]. These sponges are known as fast-growing species [53] with partially investigated life cycles [54,55]. Some species of *Mycale* have been adapted for laboratory cultivation [8,56], as well as marine farming [57]. For example, the development of aquaculture of *M. hentscheli* over seven years and through three generations of cultivars has been conducted in the New Zealand [58,59]. Furthermore, aquaculture methods based on the sexual reproduction of the demosponge *M. phyllophila* have been established in the Dongshan Bay (Fujian, China) [60] where the reproductive activity of the sponge lasted for 5–6 months and peaked when the average water temperature was above 25 °C. 

**Figure 1:**
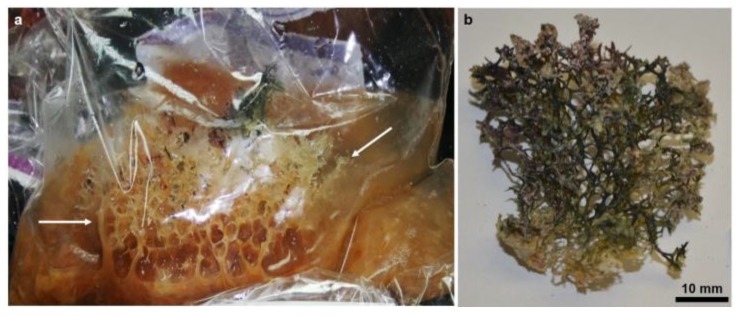
Specimen of *Mycale euplectellioides* as collected by scuba diving. After cutting the sponge from the basal part underwater, it starts to lose the outer soft mucous tissue from the hard internal skeleton. As a result, a very mucous and viscous mass appears at the bottom of the collection bag leaving the hard internal skeleton (arrows, **a**). Finally, only greenish-brown skeletal fragments can be isolated in the laboratory from the collection bags (**b**).

Thus, after preliminary experiments with respect to identification of chitin, we focused our attention on the sponge *Mycale euplectellioides* [61] reported only from the northern part of the Red Sea [62] (Figure 1).

**Figure 2:**
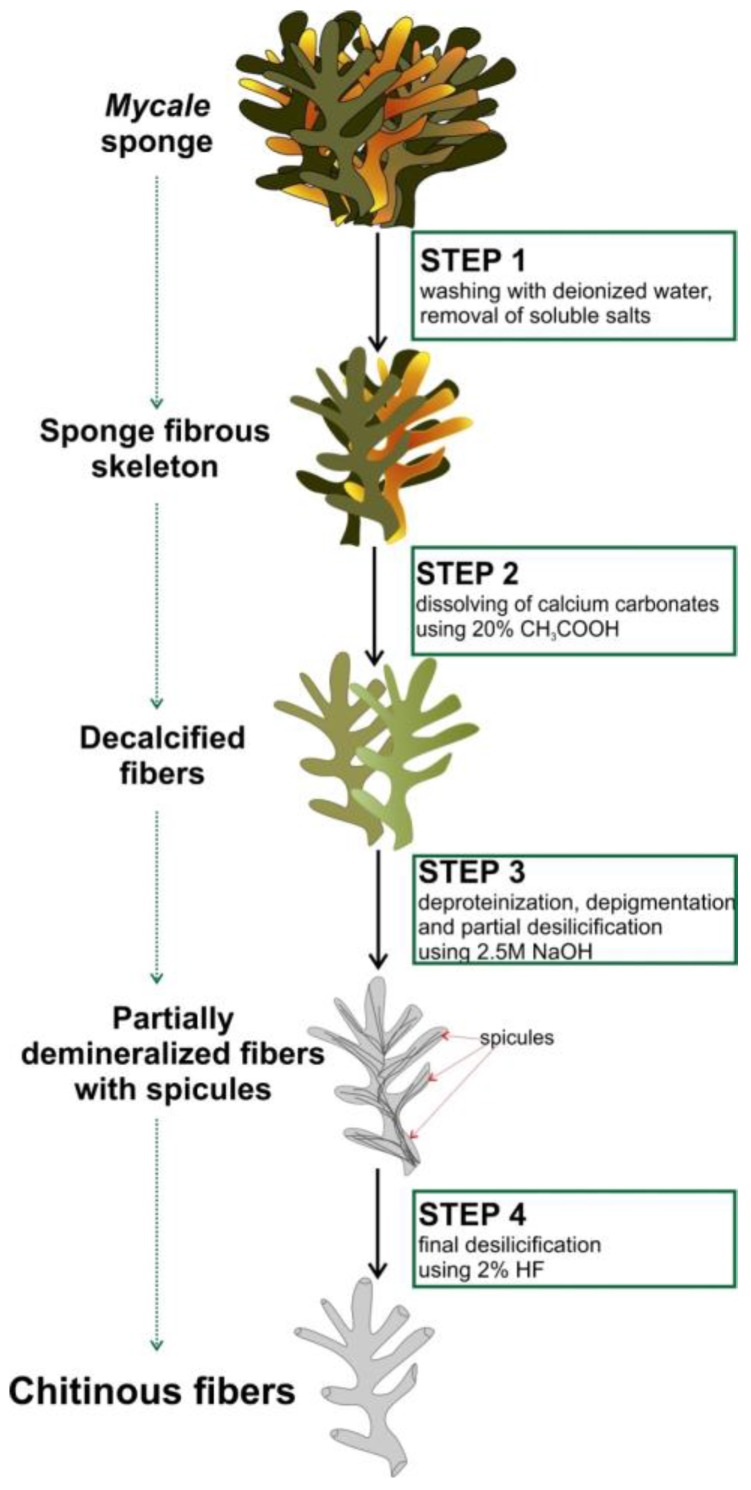
Step-by-step isolation scheme of chitinous fibers from the skeleton of the marine demosponge *M. euplectellioides*.

Here, we represent the first study on isolation and purification of chitin from the skeleton of *M. euplectellioides* demosponge according to the stepwise procedure (Figure 2) and identification of this polysaccharide using corresponding bioanalytical methods.

## 2. Results

### 2.1. Morphology and Structural Peculiarities of Organic Scaffold Isolated from M. euplectellioides Skeleton

**Figure 3:**
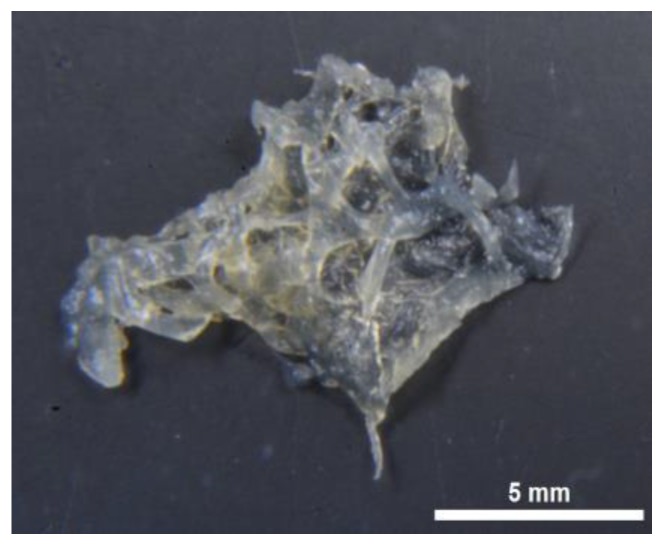
Spicule-free, colorless 3D scaffold obtained from *M. euplectellioides* according to the isolation procedure represented in Figure 2. Microstructural features of selected fibers are well visible in Figures 5 and 7b,d,e.

Chitin, in contrast to diverse pigments, lipids and proteins remain stable after treatment in both alkali-based solution (i.e., 5% NaOH) and hydrofluoric acid (HF) up to concentration of 40% at temperatures between 25 °C and 40 °C. Alternatively to spicule-free chitin-based skeletons of marine demosponges related to the order Verongiida, representatives of the genus *Mycale* (order Poecilosclerida) are rich on siliceous spicules. They also contain structural scleroprotein spongin which, however, is quickly dissolved in 2.5 M NaOH solutions. Consequently, the step-by-step treatment procedure shown in Figure 2 lead to isolation of protein- and spicules-free scaffold with 3D architecture (Figure 3) remaining to be structurally stable.

**Figure 4:**
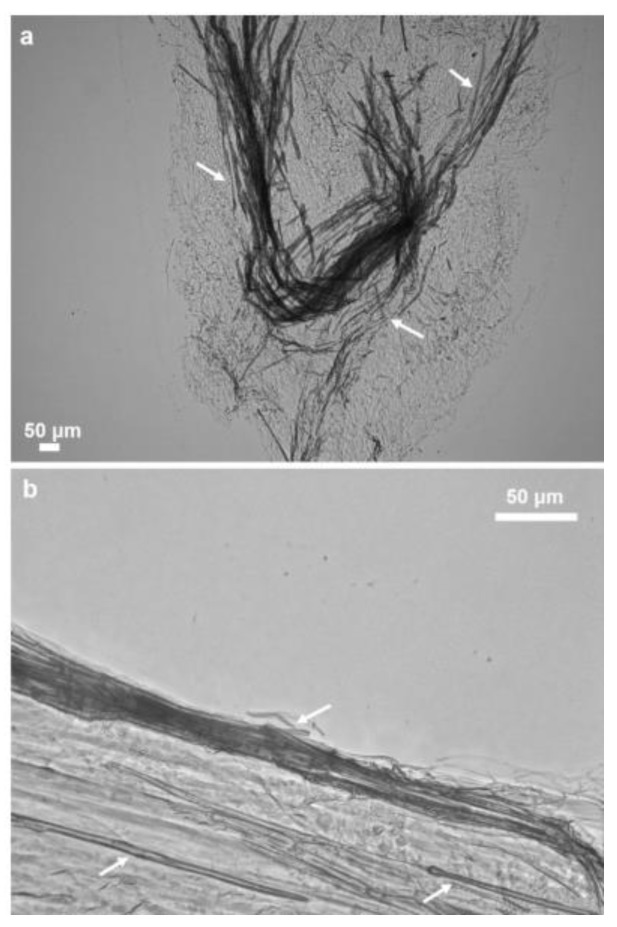
Remaining spicules (arrows in **a**,**b**) within partially demineralized skeletal fragments of *M. euplectellioides* after treatment with acetic acid and alkali. For details see Section 4.

**Figure 5:**
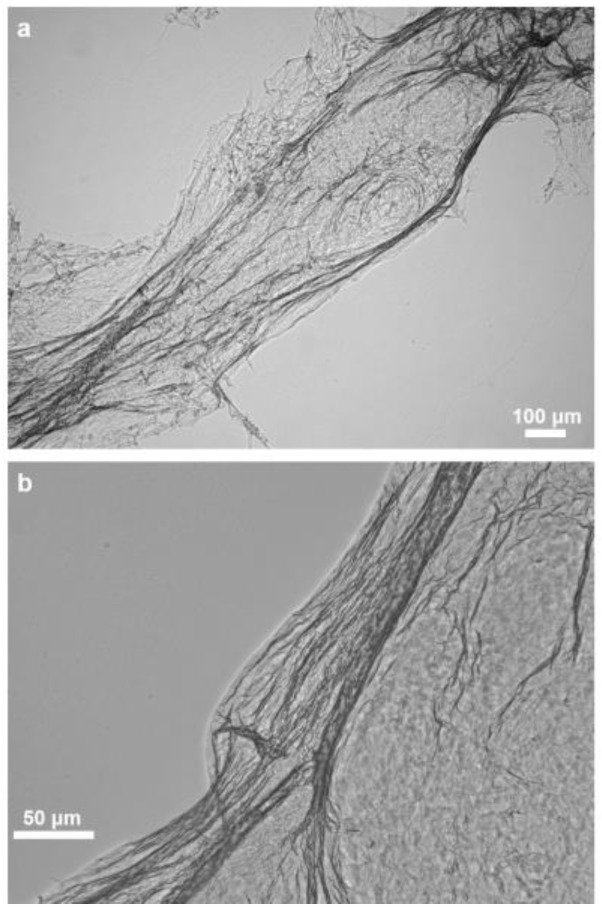
Skeletal fibers of *M. euplectellioides* after hydrofluoric acid (HF)-based treatment showing no evidence for the presence of siliceous spicules.

The isolated scaffold proves that the applied chemical treatment steps lead to the isolation of morphologically defined three-dimensional tubular construct composed of microfibers which exhibit a hollow, pipe-like, and translucent structure (Figure 3). The presented image also shows that the overall shape and morphology of the extracted skeletons closely resemble the original shape and morphology of the *M. euplectellioides* sponge (see Figure 1b). This means the extraction procedure does not lead to a breakdown of the—sometimes very fragile—demosponge structures even after supporting spicules (Figure 4) have been dissolved after treatment of the scaffold with HF-based solution (Figure 5).

**Figure 6:**
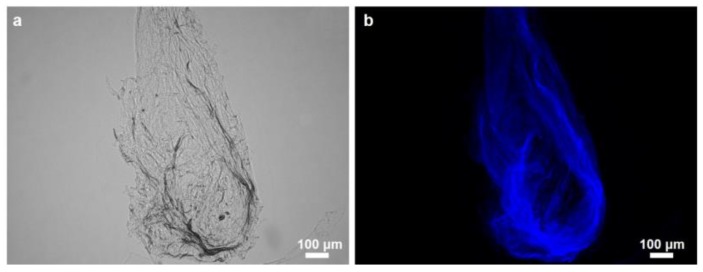
Purified skeletal fibers of *M. euplectellioides* in light (**a**) and fluorescence (**b**) microscopy modus. Very intensive fluorescence (light exposure time1/4800 s) (**b**) after Calcofluor White (CFW) staining for chitin confirms the chitinous nature of the sponge skeleton.

Structural integrity of the isolated scaffold is well visible under light and fluorescence microscopy (Figure 6), as well as using scanning electron microscopy (SEM). However, the microfibers are not so densely packed as in the case of multilayered chitin microfibers observed in diverse verongiid sponges [4,5,7,8,63]. The morphology of *M. euplectellioides* fibers is similar to that observed in chitinous fibers of the fresh water demosponge *Spongilla lacustris* [19]. This spicule-producing sponge belongs to the Spongillida and not to the order Verongiida.

**Figure 7:**
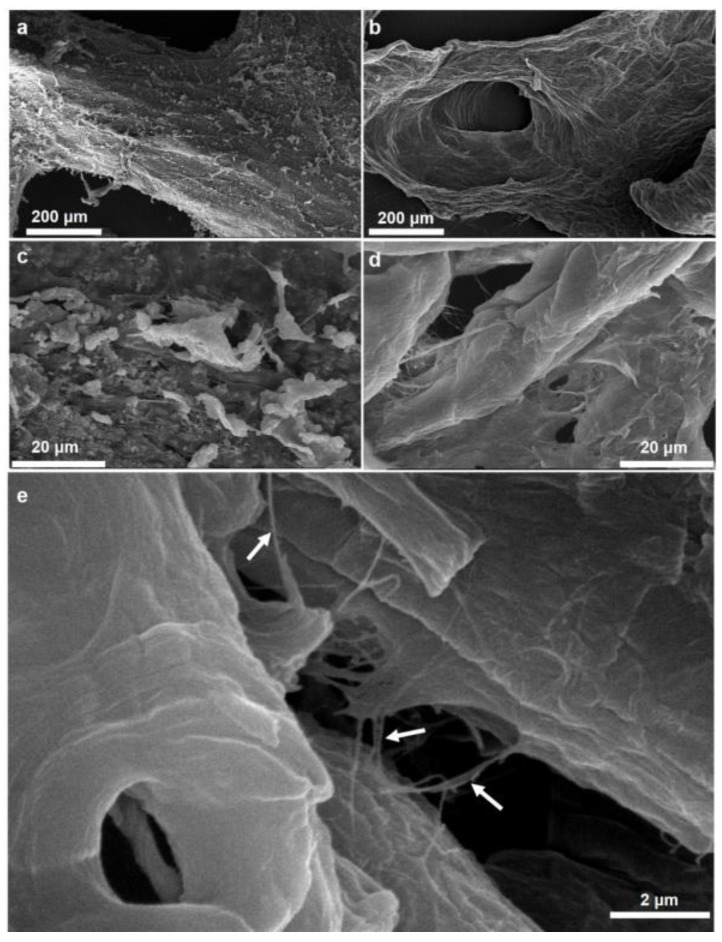
Scanning electron microscopy (SEM) imagery of the purified *M. euplectellioides* skeleton prior (**a**,**c**) and after demineralization procedure (**b**,**d**,**e**). The demineralized sample showed the nanofibrillar organization (arrows) of the fibers.

Investigations using SEM confirm with strong evidence the nanofibrillar structure of the isolated scaffold (Figure 7). Additionally, performed energy-dispersive X-ray spectroscopy (EDX) analysis (Supplementary Material) shown that isolated material is free from inorganic impurities (e.g., Ca, Mg, P, Si) and it confirms effectiveness of proposed isolation method. 

### 2.2. Identification of Chitin

Traditionally, CFW staining of demineralized skeletons of sponges which are still preserved after alkali-based treatment is the first step in the series of analytical methods used for chitin identification. Fluorescence microscopy analysis of the *M. euplectellioides* scaffold displays very strong fluorescence even under light exposure time of 1/4800 s (Figure 6). Similar results have been obtained previously for all chitin structures isolated from both demosponges and glass sponges [5,6,7,8,28,64]. To obtain more information about what kind of chitin isomorph is present in the sponge under our study, we carried out spectroscopic investigations using FTIR. 

**Figure 8:**
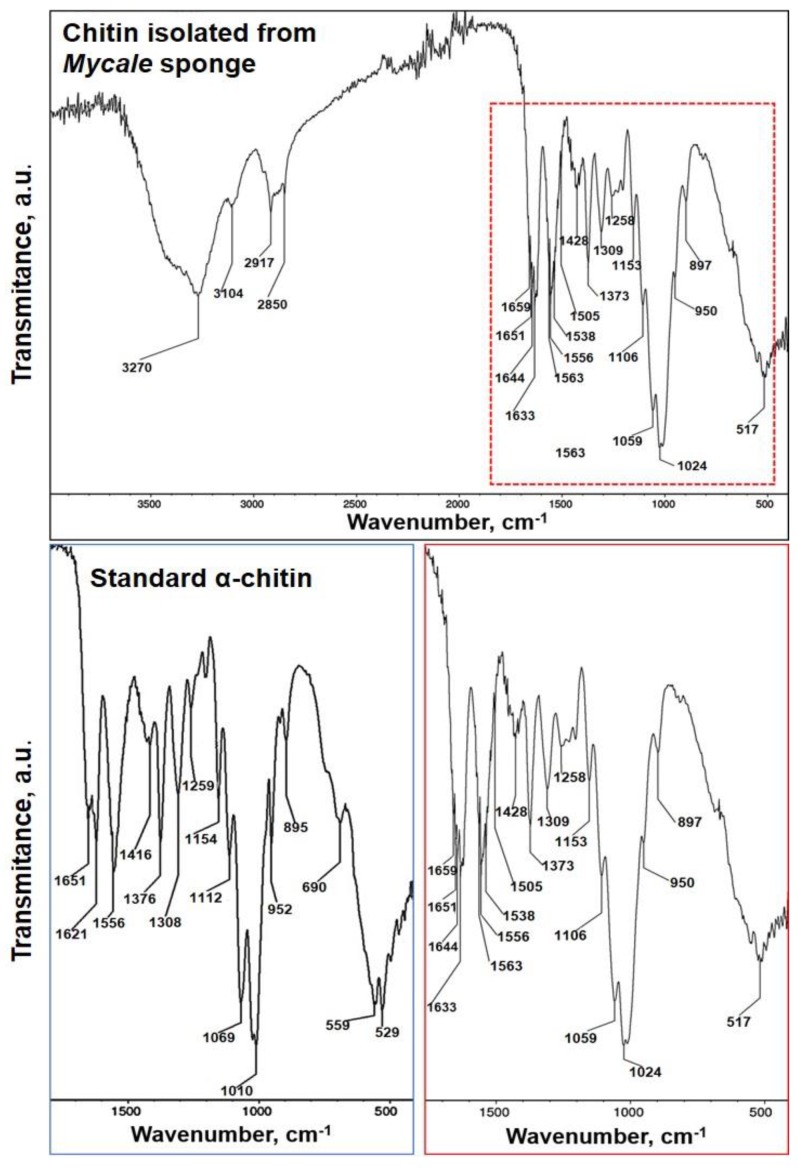
FTIR spectra of chitin isolated from *M. euplectellioides* demosponge in comparison with that of *α*-chitin standard.

FTIR spectra of the purified chitinous scaffold of *M. euplectellioides* (Figure 8) were compared with that of *α*-chitin standard. The region of the amide moiety, between 1700 and 1500 cm^−1^, yields different signatures for chitin polymorphs [65]. In this region, the spectrum of the matrix isolated from *M. euplectellioides* shows a strong adsorption band associated with the stretching vibrations of C=O group characteristic for the amide band I. The characteristic for α-chitin stretching vibrations arise from the intermolecular C=O⋯H–N and C=O⋯HO–CH_2_ hydrogen bonds, which split the amide band I split two peaks at 1659 cm^−1^ and 1633 cm^−1^, respectively [66]. Another feature, the characteristic intense band at *ν*_max_ 950 cm^−1^ assigned to γCHx is observed in both α-chitin standard and chitin isolated from *M. euplectelloides*. Additionally, the α-chitin indicative band assigned to a β-glycosidic bond is observed at *ν*_max_ 897 cm^−1^ in the spectrum of the scaffold isolated from *M. euplectelloides*. It is worth to highlight that in registered spectrum of *M. euplectelloides*, the characteristic bands for CaCO_3_ (855–876 cm^−1^) and SiO_2_ (720 cm^−1^) were not observed, confirming high purity of isolated chitin.

**Figure 9:**
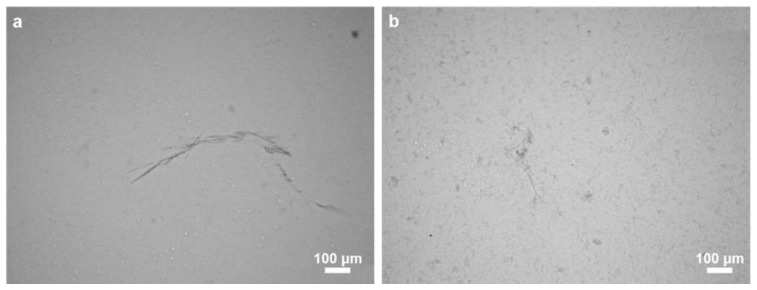
Chitinase digestion of purified and completely demineralized skeletal fiber isolated from *M. euplectellioides*. Initial stage (**a**) and the same fragment after 5 h treatment with chitinase (**b**).

Chitinases possess the ability to degrade chitin directly to low molecular weight chitooligomers including *N*-acetyl-d-glucosamine (GlcNAc). Consequently, this kind of enzymatic treatment resulted in the loss of chitin integrity and the release of residual chitin microfibers of steadily decreasing size [17]. The activity of chitinase is clearly visible using an optical microscope (Figure 9). This result from the chitinase digestion test confirms the chitinuous nature of the isolated *M. euplectellioides* scaffolds.

The Morgan-Elson assay was used as a precise method to quantify the GlcNAc released after chitinase treatment. Determination of GlcNAc in chitin-based scaffolds of *M. euplectellioides* showed 700 ± 1.5 µg *N*-acetyl-glucosamine per mg of alkali-resistant skeleton residues of this sponge. This result is similar to that reported for *S. lacustris* chitin [19]. This corresponds to approximately 70% of chitin in the dry weight of the whole sponge skeleton.

**Figure 10:**
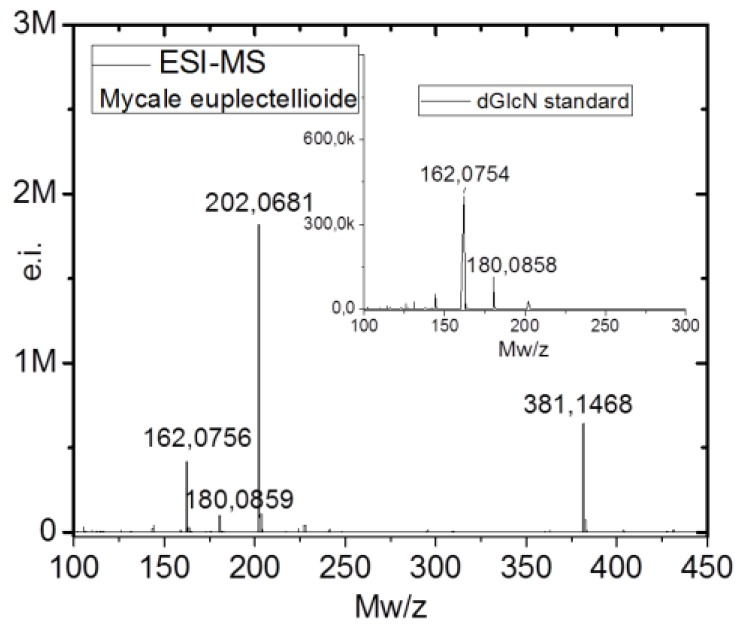
Electrospray-ionization mass spectroscopy (ESI-MS) investigation of the chitin isolated from the skeletal scaffold of *M. euplectellioides*. Insertion is the ESI-MS spectra of commercial (dGlcN) standard for comparison.

Additionally, electrospray-ionization mass spectroscopy (ESI-MS) measurements were used to identify the presence of chitin. Acetic hydrolysis of chitin resulted in the formation of d-glucosamine (dGlcN), which can be easily identified by the ESI-MS spectroscopy. This method is a standard for chitin identification and was used for chitin visualization in complex organisms [21,64] and even in 505-million-year-old chitin-containing fossil remains [28]. The ESI-MS spectrum of the *M. euplectellioides* hydrolyzed skeletal scaffold revealed four main ion peaks at *m*/*z* 162.08, 180.09, 202.07 and 381,15 (Figure 10). The ion peaks at *m*/*z* 162.08, and 180.09 are identical to the peaks in the ESI-MS spectrum of the commercial (dGlcN) standard (Figure 10 insert). The ion peak at *m*/*z* 180.9 is equivalent to the [M + H]^+^ of dGlcN molecule, while the ion peak at 161.85 is equivalent to dGlcN after loss of one molecule of H_2_O [M − H_2_O + H]^+^. The week ion peaks at *m*/*z* 202.07 and 381.15 are corresponding to [M + K]^+^ and [2M + K]^+^ species, respectively, which represent the K-bound-dGlcN monomer and noncovalent dimer correspondingly. 

## 3. Discussion

The obtained results showed that in contrast to non-spiculated demosponges of the Verongiida order, the chitin isolation procedure (see Figure 2) is more complex in the case of *M. eplectelloides*. The morphology of isolated chitinous fibers differs from tube-like, multilayered skeletal architecture known in verongiid sponges [5,7,8]. It is well recognized that secondary metabolites of verongiids as bromotyrosines are inhibitors of microbial chitinases [7,8]. Thus, a biological function of these compounds for survival of verongiid sponges can be suggested. What is the situation with secondary metabolites within the genus *Mycale* and their relationship with the skeletal chitin?

Sponges of the genus *Mycale* are, probably, among the richest sources of pharmacologically active compounds isolated from marine organisms [67,68,69]. Such secondary metabolites as pateamines [70], mycalolides [71] as well as mycalamide A and D [72,73] are known to be extremely cytotoxic [74,75,76]. Mycalamide A and B also showed antiviral, antitumor [77,78] and antibacterial [79] features. Dihydroxymycalolide A isolated from *M. izuensis* was cytotoxic against HeLa cells with an IC_50_ value of 2.6 ng/mL [80]. Interestingly, the same sponge species are able to synthetize so-called azumamides, which are related to cyclic tetrapeptides with histone deacetylase inhibitory activity [81,82]. Secomycalolide A has been described as a proteasome inhibitor [83]. Peloruside A, from, *M. hentscheli*, possesses anti-mitotic properties with paclitaxel-like microtubule-stabilizing activity [70,84,85,86], and has shown potent antiproliferative activity in cancer cell lines in addition to its inhibitory effects on tumor growth in mouse models [87,88]. Peloruside B, have been reported as a potent antitumor macrolide [89]. Lipophilic 2,5-disubstituted pyrroles isolated from a *Mycale* sp. inhibited hypoxia-induced factors HIF with moderate potency (IC_50_ values < 10 μM) [90]. These compounds appear to disrupt mitochondrial reactive oxygen species (mROS) regulated HIF-1 signaling under hypoxic conditions. The antidiabetic activities of some octapyrroles from *M. mytilorum* [91] and 5-alkylpyrrole-2-carboxaldehyde derivatives from the South China Sea sponge *M. lissochela* [92] are reported. Data about synthesis of diverse steroids by *Mycale* are reported in the literature [93,94]. New steroidal lactone named mycalone has been isolated from an Australian *Mycale* species [95]. New steroidal oligoglycosides, mycalosides, have been isolated from the polar extract of the Caribbean sponge *M. laxissima* [96]. These compounds inhibited the fertilization of eggs by sperm of the sea urchin *Strongylocentrotus nudus* preincubated with these mycalosides.

To our best knowledge, there are only few reports on the chemistry of the Red Sea sponge *M. euplectellioides*. New fatty acids related to hexacosa-(6*Z*,10*Z*)-dienoic acid methyl ester and hexacosa-(6*Z*,10*Z*)-dienoic acid with weak activity against A549 non-small cell lung cancer, the U373 glioblastoma and the PC-3 prostate cancer cell lines have been described recently in the research group of Youssef [97]. In addition, new ceramides have been isolated from the methanolic extracts of this demosponge. These compounds were proposed as promising lead ceramides for the discovery and design of potent anti-choline esterase drug candidates, which would be used for Alzheimer disease eradication [98]. The possible inhibitory activity of the compounds produced by diverse *Mycale* species as listed above against chitinases of microbial origin is unknown.

**Figure 11:**
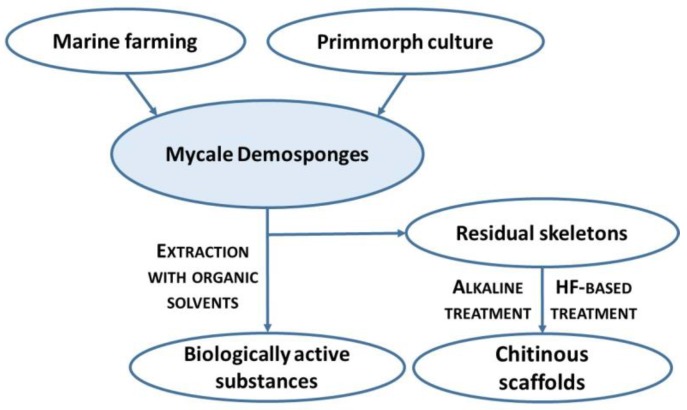
Schematic view of the possible uses of *Mycale* sponges.

Until now, diverse secondary metabolites from members of the genus *Mycale* have been purified using traditional organic solvent-based extraction approaches. There are no reports on isolation methods for these metabolites which are based on treatment with alkaline solutions, as well as about structural stability of such biomacromolecules at alkaline pH levels. Experiments with bromotyrosine- and chitin-producing demosponges represented by the order Verongiida showed that bromotyrosines and chitin-based scaffolds could be isolated from the sponge skeletons using a stepwise extraction procedure mainly based on the use of NaOH [6]. Recently, a patented method for isolation of both bromotyrosines and chitinous skeletal frameworks from selected sponges without disruption of the skeletons in the mortar (this being the traditional procedure for extraction of bromotyrosines) has been proposed [99]. Here, we propose the schematic view of the principal steps which can be now applied for purification of secondary metabolites and chitin from the *Mycale* sponges (Figure 11).

There are no doubts about the necessity of the development of novel, more effective methodologies for extraction of biologically active compounds together with chitinous scaffolds from *Mycale* demosponges. In particular, *Mycale* species, which are already adapted for cultivation under marine ranching conditions [58,60], showed high potential in this case.

We suggest that the discovery of chitin within other representatives of the genus *Mycale* would be the next step in the evaluation of the possibility to accept these worldwide distributed sponges as novel renewable source for both biologically active metabolites and chitin which are perspective for pharmacology and biomaterials oriented biomedicine, respectively.

## 4. Materials and Methods 

### 4.1. Collection of the Samples

The specimens of *Mycale euplectellioides* (Row, 1911) (Demospongiae: Poecilosclerida: Mycalidae) were collected by scuba diving at depths of 7–10 m in Red Sea 20 km south of Hurghada, Egypt (N 27°02’46.8’’ E 33°54’21.4’’), in July 2017. Originally, the sponge composed of 15–30 cm hollow reddish tubes―connected together at a basal part attached to a hard substrate (rock). The tubes were hollow and measured about 7–10 cm at the apical part. When the sponge cut with a knife underwater, it exposes reddish mucous-like material (Figure 1).

### 4.2. Isolation of Chitin from M. euplectellioides

Isolation of the chitin-based skeletal fibers from *M. euplectellioides* was achieved in several steps (Figure 2). Freeze-dried skeletons of the sponge *M. euplectellioides* (Figure 1b) were washed three times with demineralized water for removal of various water-soluble impurities including salts. The washed samples were placed into glassy Petri dishes and cut into 1.5 × 2 cm large fragments. Highly visible greenish-colored and mechanically rigid skeletal fragments have been decalcified at room temperature using 20% acetic acid during 4 h and rinsed in distilled water up to pH 6.8 (Figure 2, Step 2). This procedure is necessary to remove possible calcium and magnesium carbonate containing contaminations (i.e., debris of crustaceans, or mollusks) within the sponge skeltone. After decalcification, the skeletal fragments were treated with 2.5 M NaOH (Sigma-Aldrich, Taufkirchen, Germany) at 37 °C for 72 h (Memmert Incubator, Schwabach, Germany) to achieve depigmentation, deproteinization, as well as partial desilicification. After the removal of residual pigmentation using multiple washing in demineralized water, colorless scaffolds were obtained (Figure 2, Step 3). Observation using stereo and light microscopy showed that glassy spicules were still present within skeletal scaffolds (Figure 4). Subsequently, the colorless skeletal fibers were placed in plastic boxes and treated with 2% HF (Sigma-Aldrich, Taufkirchen, Germany) during 24 h at room temperature for complete desilicification (Figure 2, step 4). Afterwards, the samples were isolated from the plastic boxes and rinsed with demineralized water up to pH 6.8. Obtained fibrous scaffolds (Figure 3) were placed into 50 mL glass bottles and stored in demineralized water at 4 °C till their use for analytical investigations with respect to further chitin identification.

### 4.3. Light and Fluorescent Microscopy Analysis and Imaging

Collected sponge samples and isolated skeletons as well as purified chitinous scaffolds of *M. euplectellioides* have been studied using stereomicroscope Di-Li (Kaiserslautern, Germany), BZ-9000 microscope (Keyence, Osaka, Japan) in light and in fluorescent microscopy modus. Photos and macroscopic close-up pictures were made using camera Nikon D-7100 with objective lenses Nikon AF-S DX 18–105 mm f/3.5–5.6 G or Nikon AF-S VR Micro-Nikkor 105 mm f/2.8G IF-ED, Tokyo, Japan). Figures were prepared using freeware software (GNU Image Manipulation Program “GIMP 2.8”).

### 4.4. Calcofluor White Staining Test

Calcofluor white (CFW) (Fluorescent Brightener M2R, Sigma-Aldrich, Taufkirchen, Germany), which shows enhanced fluorescence when it binds to polysaccharides, especially chitin, was used. The fragments of natural and demineralized sponge skeleton samples were placed in 0.1 M Tris–HCl buffer (pH 8.5) for 30 min. Afterwards, the samples were stained using 0.1% CFW solution for 2 h in darkness, rinsed several times with demineralized water, dried at room temperature during 5 h and analyzed using fluorescent microscopy.

### 4.5. Scanning Electron Microscopy Analysis

The surface morphology and microstructure of the isolated *M. euplectellioides* skeletal fragments (Figure 1b) as well as isolated scaffolds (Figure 3) were analyzed using ESEM XL 30 Scanning Electron Microscope (Philips, Eindhoven, The Netherlands). Prior the examination, samples were fixed in a sample holder and covered with a carbon layer for 1 min using an Edwards S150B sputter coater. 

### 4.6. Chitinase Digestion Test

Yatalase® from culture supernatants of *Corynebacterium* sp. OZ-21 (Cosmo Bio, Tokyo, Japan) was used for this test. Yatalase is a complex enzyme, consists mainly of chitinase, chitobiase and β-1,3-glucanase. One unit of this enzyme released 1 μmol of *N*-acetylglucosamine from 0.5% chitin solution and 1 μmol of p-nitrophenol from p-nitrophenyl-*N*-acetyl-β-D-glucosaminide solution in 1 min at 37 °C and pH 6.0. The selected, completely demineralized scaffolds of *M. euplectellioides* (Figure 3) were incubated in enzyme solution containing 10 mg/mL Yatalase dissolved in citrate phosphate buffer at pH 5.0 for 5 h. The progress of digestion was monitored under light microscopy using BZ-9000 microscope (Keyence, Osaka, Japan).

### 4.7. Estimation of N-acetyl-d-glucosamine (NAG) Contents and Electrospray Ionization Mass Spectrometry 

The Morgan-Elson assay was used to quantify the *N*-acetyl-d-glucosamine released after chitinase treatment. Purified and dried *M. eplecteoides* chitin (6 mg) was pulverized to a fine powder in an agate mortar. The samples were suspended in 400 mL of 0.2 M phosphate buffer at pH 6.5. A positive control was prepared by solubilizing 0.3% colloidal chitin in the same buffer. Equal amounts (1 mg/mL) of three chitinases (EC 3.2.1.14 and EC 3.2.1.30): *N*-acetyl-d-glucosaminidase from *Trichoderma viride* (Sigma-Aldrich, No. C-8241), and two poly (1,4-a-(2-acetamido-2-deoxy-d-glucoside)) glycanohydrolases from *Serratia marcescens* (Sigma-Aldrich, No. C-7809), and *Streptomyces griseus* (Sigma-Aldrich, No. C-6137), respectively, were suspended in 100 mM sodium phosphate buffer at pH 6.0. Digestion was started by mixing 400 mL of the samples and 400 mL of the chitinase mix. Incubation was performed at 37 °C. The reaction was stopped after 114 h by adding 400 mL of 1% NaOH, followed by boiling for 5 min. The vessels were centrifuged at 7000 rpm for 5 min and the products analyzed using the 3,5-dinitrosalicylic acid assay (DNS). For this purpose, 250 ml of the supernatants and 250 mL of 1% DNS were dissolved in a solution containing 30% sodium potassium tartrate in 0.4 M NaOH, mixed and incubated for 5 min in a boiling water bath. Thereafter, the absorbance at 540 nm was recorded using a Tecan Spectrafluor Plus Instrument (Mannedorf/Zurich, Switzerland). Data were interpolated into a standard curve via the serial dilution (0–3.0 mM) of *N*-acetyl-d-glucosamine (Sigma-Aldrich, No. A-8625) and DNS. A sample which contained chitinase solution without substrate was used as control.

Sample preparation for ESI-MS: specimens obtained after HF treatment (Figure 3 and Figure 5) were hydrolyzed in 6 M HCl for 24 h at 50 °C. After the HCl hydrolysis the samples were filtrated with 0.4 µm filter and freeze-dried in order to remove excess HCl. The remaining solid was dissolved in water for ESI-MS analysis. Standard d-glucosamine was purchased from Sigma-Aldrich (Taufkirchen, Germany). All ESI-MS measurements were performed on Waters TQ Detector ACQUITYuplc mass spectrometer (Waters, Wilmslow, UK) equipped with ACQUITYuplc pump (Waters, Wilmslow, UK) and BEHC18 1.7 mm 2.1 × 50 mm UPLC column. Nitrogen was used as nebulizing and desolvation gas. Graphs were generated using Origin 8.5 for PC.

### 4.8. FTIR Spectroscopy

Transmission spectra for isolated scaffolds and α-chitin (as reference sample) were measured with the spectral resolution of 4 cm^−1^ using a FTIR spectrometer TENSOR 27 (Bruker, Mannheim, Germany). α-Chitin standard was obtained from INTIB GmbH (Freiberg, Germany).

## 5. Conclusions

Marine demosponges of the genus *Mycale* seem to represent a gold mine for marine pharmacology, marine biotechnology, as well as for marine-bioinspired materials science. Their high potential for many applications is due to their ability to grow under marine farming conditions and to synthetize a broad variety of secondary metabolites with antiviral, antibiotic, antidiabetic, cytotoxic and antitumor activities, as well as chitin. Here, we showed for the first time that chitin is present as a structural component in skeletons of the Red Sea demosponge *M. euplectelloides*. The question of chitin synthesis among members of the genus *Mycale* should gain importance as a result of our findings. Consequently, the evolution, localization and functions of chitin within the demosponge *M. euplectelloides* as well as in other representatives of the family Mycalidae should now be examined. Further investigations on detailed structural features of chitin from *M. euplectelloides* using solid state NMR, X-ray diffraction (XRD), high resolution transmission electron microscopy (HRTEM) and electron diffraction are in progress now and will be presented in a separate bioanalytical publication. Additionally, separate studies should be carried out to identify chitin synthase genes within the genomes of diverse representatives of the *Mycale* genus. Also, additional investigations are necessary to obtain understanding of the nature and origin of spicules-containing skeletons of these demosponges. It is still not clear how much spongin and chitin domains are present in them. Novel approaches must be proposed which will bring together modern bioanalytical and molecular biology methods for better understanding of the poriferan chitins synthesis in diverse taxa on the molecular level. The best way to solve this challenging task is a coherent synergetic collaboration of spongologists together with experts in marine chemistry, pharmacology, marine biology, marine biotechnology and biomaterials science using their multidisciplinary knowledge and experiences.

## Supplementary Materials

The following is available online: http://www.mdpi.com/1660-3397/16/2/68/s1. Figure S1. EDX spectrum of chitin isolated from *M. euplectellioides* demosponge. Spectrum was registered with use of a XL 30 ESEM Philips-Scanning Electron Microscope (Netherlands). The uncoated samples were investigated at an accelerating voltage of 20 kV.
