# Supplementary material for: First Report on Chitin in a Non-Verongiid Marine Demosponge: The Mycale euplectellioides Case
Source: Mar Drugs. 2018 Feb 20;16(2):68. doi: 10.3390/md16020068 (PMC5852496; doi:10.3390/md16020068)
Supplement: Supplementary file 1 [file marinedrugs-16-00068-s001.pdf]

marine drugs

Supplementary information

First Report on Chitin in a Non-Verongiid Marine Demosponge: The *Mycale euplectellioides* Case.

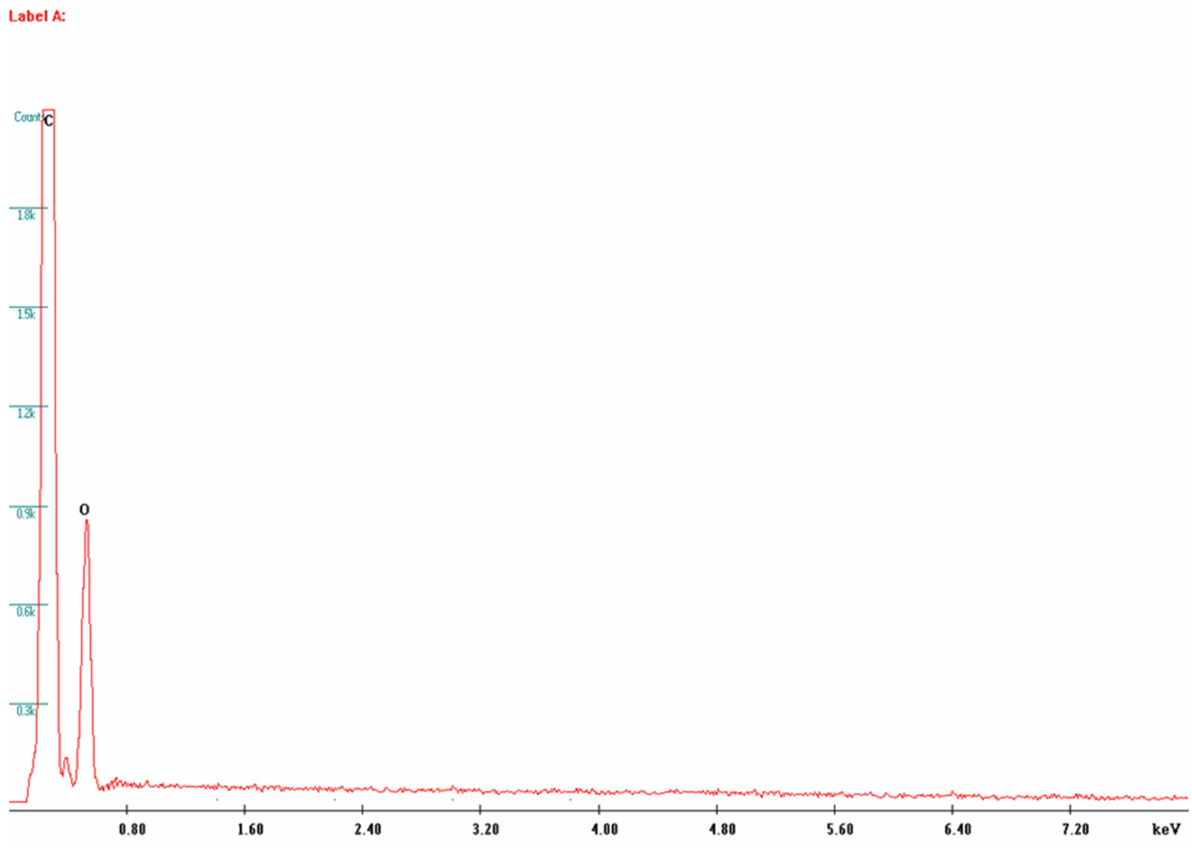

Figure S1. EDX spectrum of chitin isolated from *M. euplectellioides* demosponge. Spectrum was registered with use of a XL 30 ESEM Philips-Scanning Electron Microscope (Netherlands). The uncoated samples were investigated at an accelerating voltage of 20 kV.
